# Ventricular Tachycardia

**DOI:** 10.21980/J8KD2R

**Published:** 2023-10-31

**Authors:** Rohit Menon, Geremiha Emerson, Jennifer Yee

**Affiliations:** * The Ohio State University, Department of Emergency Medicine, Columbus, OH

## Abstract

**Audience:**

This scenario was developed to educate emergency medicine residents on the diagnosis and management of ventricular tachycardia (VT) that is refractory to single dose anti-arrhythmic management.

**Background:**

Electrical storm, defined as three or more episodes of sustained VT, ventricular fibrillation, or appropriate shocks from an implantable cardioverter defibrillator within 24 hours,^1^ has a mortality rate up to 14% in the first 48 hours.^2^ Ventricular tachycardia may present in a heterogenous fashion, not only with stable versus unstable clinical presentations, but also with different electrocardiographic morphologies and etiologies.^1^ Understanding how to rapidly diagnose, treat, and utilize second or third-line treatments is vital in the setting of refractory ventricular tachycardia rather than relying on the success of first-line agents. Appreciation for what medications are readily available in your crash cart and medication dispensing cabinet is critical for timely management for refractory ventricular tachycardia.

**Educational Objectives:**

At the conclusion of the simulation session, learners will be able to: 1) identify the different etiologies of VT, including structural heart disease, acute ischemia, and acquired or congenital QT syndrome; 2) describe confounding factors of VT, such as electrolyte abnormalities and sympathetic surge; 3) describe how to troubleshoot an unsuccessful synchronized cardioversion, including checking equipment connections, increasing delivered energy, and changing pad placement; 4) compare and contrast treatments of VT based on suspected underlying etiology; 5) describe reasons to activate the cardiac catheterization lab other than occlusive myocardial infarction; and 6) identify appropriate disposition of the patient to the cardiac catheterization lab.

**Educational Methods:**

This session was conducted using high-fidelity simulation, followed by a debriefing session and lecture on the diagnosis, differential diagnosis, and management of VT. Debriefing methods may be left to the discretion of participants, but the authors have utilized advocacy-inquiry techniques. This scenario may also be run as an oral board case.

**Research Methods:**

Our residents are provided a survey at the completion of the debriefing session so they may rate different aspects of the simulation, as well as provide qualitative feedback on the scenario.

**Results:**

The local institution’s simulation center’s electronic feedback form is based on the Center of Medical Simulation’s Debriefing Assessment for Simulation in Healthcare (DASH) Student Version Short Form^3^ with the inclusion of required qualitative feedback if an element was scored less than a 6 or 7. Twelve learners completed a feedback form. This session received 6 and 7 scores (consistently effective/very good and extremely effective/outstanding, respectively) other than three isolated 5 scores. The lowest average score was 6.67 for “Before the simulation, the instructor set the stage for an engaging learning experience.” The highest average score was 7 for “The instructor helped me see how to improve or how to sustain good performance.” The form also includes an area for general feedback about the case at the end. Illustrative examples of feedback include: “Excellent care and debrief.” Specific scores are available upon request.

**Discussion:**

This is a cost-effective method for reviewing VT diagnosis and management. The case may be modified for appropriate audiences, such as describing what medications may be readily available in a free-standing emergency department or pre-hospital setting.

**Topics:**

Medical simulation, ventricular tachycardia, cardiac emergencies, dysrhythmias, cardiology, emergency medicine.

## USER GUIDE

[Table t1-jetem-8-4-s25]

**Table t1-jetem-8-4-s25:** 

List of Resources:	
Abstract	25
User Guide	27
Instructor Materials	29
Operator Materials	39
Debriefing and Evaluation Pearls	41
Simulation Assessment	44

**Learner Audience:**

Interns, Junior Residents, Senior Residents

**Time Required for Implementation:**

**Instructor Preparation:** 30 minutes

**Time for case:** 20 minutes

**Time for debriefing:** 40 minutes

**Recommended Number of Learners per Instructor:**

3–4

**Topics:**

Medical simulation, ventricular tachycardia, cardiac emergencies, dysrhythmias, cardiology, emergency medicine.

**Objectives:**

By the end of this simulation session, the learner will be able to:

1. Identify the different etiologies of VT, including structural heart disease, acute ischemia, and acquired or congenital QT syndrome.
2. Describe confounding factors of VT, such as electrolyte abnormalities and sympathetic surge.
3. Describe how to troubleshoot an unsuccessful synchronized cardioversion, including checking equipment connections, increasing delivered energy, and changing pad placement.
4. Compare and contrast treatments of VT based on suspected underlying etiology.
5. Describe reasons to activate the cardiac catheterization lab other than occlusive myocardial infarction.
6. Identify appropriate disposition of the patient to the cardiac catheterization lab.

### Linked objectives and methods

Patients with ventricular tachycardia require prompt recognition and treatment in order to restore sinus rhythm and possibly prevent unstable tachycardia or a pulseless state. After this scenario, providers will be able to describe the different causes of ventricular tachycardia (objective 1) as well as confounding factors (objective 2). Providers will also be able to troubleshoot an unsuccessful synchronized cardioversion (objective 3), as well as compare treatments depending on suspected underlying etiology (objective 4). Other reasons for activating the cardiac catheterization lab may be described (objective 5), and providers will appropriately disposition the patient to the catheterization lab (objective 6). Objectives were tracked by facilitators taking notes during the simulation scenario for the subsequent debriefing discussion.

This simulation scenario allows learners to reinforce VT diagnostic and management skills in a psychologically safe learning environment, and then receive formative feedback on their performance.

### Recommended pre-reading for instructor

We recommend that instructors review literature regarding VT, including presenting signs/symptoms, diagnosis, and management. Suggested readings include materials listed under the “References/suggestions for further reading” section below.

### Results and tips for successful implementation

This simulation was written to be performed as a high-fidelity simulation scenario, but also may be used as a mock oral board case.

The case was written for emergency medicine residents.

The case started off with an elderly gentleman complaining of chest discomfort. Most groups promptly placed the patient on the monitor and quickly identified ventricular tachycardia as the likely source of his symptoms. One group anchored on to the complaint of generalized weakness and began a general assessment for an acute stroke.

This case was challenging due to the number of treatments that needed to be initiated. Groups could precipitate ventricular fibrillation by administering incorrect medications, defibrillating instead of administering a synchronized cardioversion, or waiting too long to start second or third-line medications. Most groups administered at least one antiarrhythmic, the most common of which was amiodarone. Multiple groups discussed starting a second-line option; however, only two groups subsequently ordered this. In terms of electrical management, all learners attempted cardioversion. If done correctly, a brief rhythm strip showing ST elevation was shown before converting back into a ventricular arrythmia. One group forgot to synchronize the defibrillator which resulted in ventricular fibrillation. All but two groups ran out of time at 8 minutes to start second-line therapy and subsequently needed to perform advanced cardiovascular life support (ACLS) for ventricular fibrillation to obtain return of spontaneous circulation (ROSC).

The case was written to necessitate use of two sympatholytic or negative inotropic medications for clinical improvement; however, it was mentioned that in a real-world setting, the diagnosis of ventricular storm would be made after three unsuccessful attempts with electrical management. One additional differential from multiple earlier groups that we noted was supraventricular tachycardia with aberrancy. We believe this to be due to recency bias from a rapid case conference that was coincidentally presented the same day a few hours before our simulation session. This was not noted when the simulation was presented the following week.

During debriefing, residents gave positive feedback regarding the review of ventricular dysrhythmias and ventricular storm. The main take away learning point was to consider underlying acute ischemia for patients with polymorphic ventricular tachycardia without a prolonged QTc. Facilitators may choose to use this challenging scenario; however, the case can be modified to more junior learners by focusing on management of Torsades de Pointes or simple monomorphic VT.

Our scenario began with the patient demonstrating significant tachycardia and progression to more evident signs of ischemic chest pain. We did not want to focus on ST-elevation myocardial infarction (STEMI) management nor post arrest care; however, facilitators may elect to edit the case to emphasize these points. Given that this was a case of polymorphic VT, it could easily be transitioned to management of Torsades de Pointes.

We found that having the patient complain more frequently about chest pain queued learners into considering acute coronary syndrome as an underlying etiology. The key element was being able to briefly show an ST elevation on the rhythm strip immediately after cardioversion. Nearly all learners obtained an electrocardiogram (ECG) after and were able to successfully identify the inferior STEMI and proceed to catheterization lab activation. If the appropriate number of interventions was not met, the confederate nurse would mention that the interventionalists are stuck in another case. Modifications to this case could be made to allow for conversion out of VT after one negative inotropic medication and two rounds of electricity. A more realistic example would require three cardioversions or defibrillations. We chose our methods to ensure the case would not be completed solely by following ACLS protocol. Near the end of the case, cardiology called the groups back to ask for a summary of the patients’ care, and the encounter was concluded.

## Supplementary Information



## Figures and Tables

**Table t2-jetem-8-4-s25:** 

#1	ECG, VT
#2	ECG, previous sinus rhythm
#3	ECG, post conversion. ST-elevation myocardial infarction
#4	Chest x-ray
#5	Complete blood count (CBC)
#6	Basic metabolic panel (BMP)
#7	Brain natriuretic peptide (BNP)
#8	Magnesium
#9	Thyroid Stimulating Hormone (TSH)
#10	Troponin

**Table t3-jetem-8-4-s25:** 

**Vitals:**	Heart Rate (HR) 160	Blood Pressure (BP) 130/70
	Respiratory Rate (RR) 20	Temperature (T) 98.0°F
	Oxygen Saturation (O_2_Sat) 98% on room air	

**Figure f1-jetem-8-4-s25:**
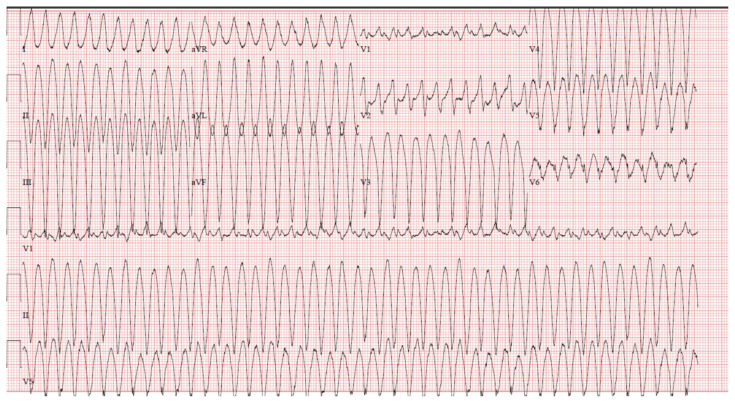


**Figure f2-jetem-8-4-s25:**
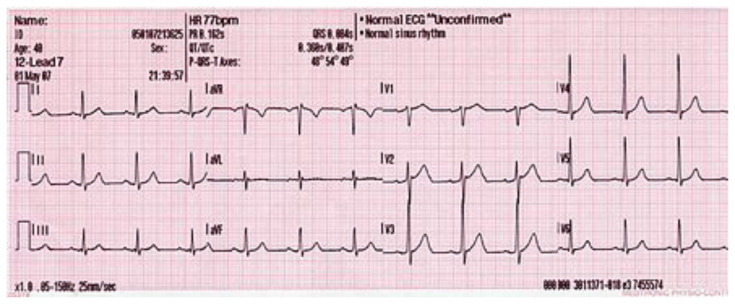


**Figure f3-jetem-8-4-s25:**
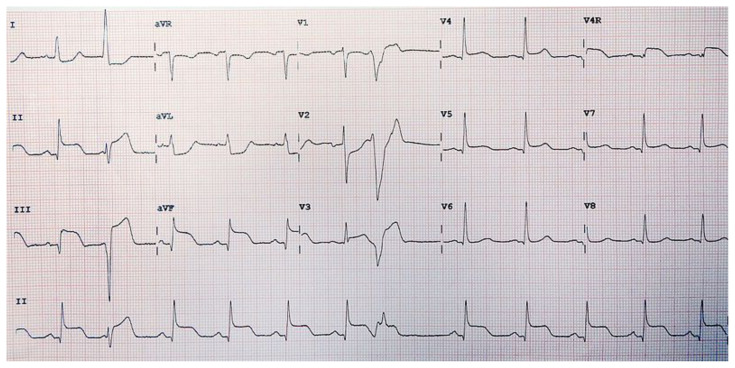


**Figure f4-jetem-8-4-s25:**
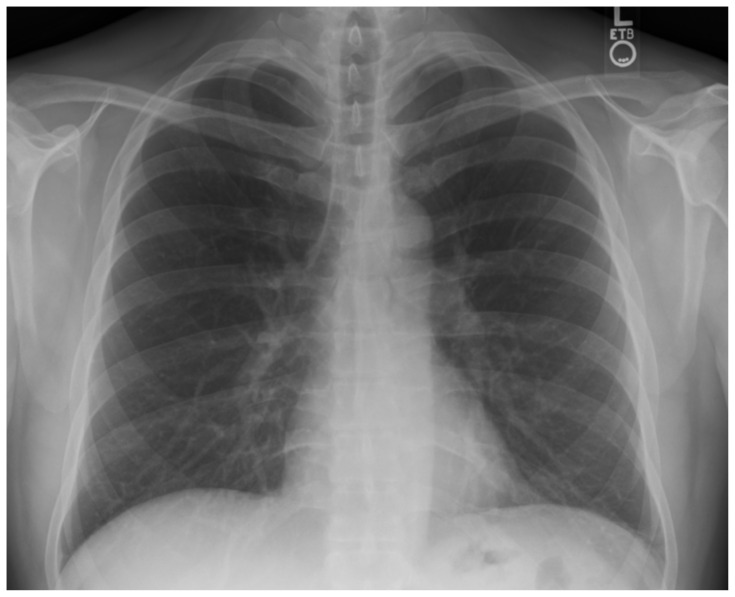


**Table t4-jetem-8-4-s25:** 

***Complete blood count (CBC)***	
White blood count (WBC)	12.2 × 1000/mm^3^
Hemoglobin (Hgb)	13.5 g/dL
Hematocrit (HCT)	34%
Platelet (Plt)	390 × 1000/mm^3^

**Table t5-jetem-8-4-s25:** 

***Basic metabolic panel (BMP)***	
Sodium	133 mEq/L
Potassium	4.5 mEq/L
Chloride	99 mEq/L
Bicarbonate (HCO_3_)	26 mEq/L
Blood Urea Nitrogen (BUN)	34 mg/dL
Creatinine (Cr)	1.0 mg/dL
Glucose	140 mg/dL
Calcium	8.0 mg/dL

**Table t6-jetem-8-4-s25:** 

***Basic natriuretic peptide***	120 pg/mL

**Table t7-jetem-8-4-s25:** 

***Magnesium***	2.0 mEq/L

**Table t8-jetem-8-4-s25:** 

***TSH***	2.30 uIU/mL (0.550–4.780 uIU/mL)

**Table t9-jetem-8-4-s25:** 

***Troponin***	3.150 ng/mL

**Table t10-jetem-8-4-s25:** SIMULATION EVENTS TABLE:

Minute (state)	Participant action/ trigger	Patient status (simulator response) & operator prompts	Monitor display (vital signs)
0:00 (Baseline)	Simulated Patient nurse brings the team into the patient’s room in the emergency department.	Participants should begin by placing the patient on a monitor, obtaining history from patient, obtaining an ECG, and performing a physical exam.	A: T 98°F HR 160 BP 130/70 RR 20 O2 sat 98% room air (RA)
3:00	Team should recognize VT on the ECG. IV placed, labs obtained, defibrillator pads placed. Labs should be ordered.	Until patient is cardioverted twice (needs to either increase energy or change pad location on second attempt) and given two different antiarrhythmics, vitals remain unchanged from vitals (B). If the team defibrillates the awake patient, go to vitals (C). If the team gives isoproterenol, go to vitals (C). If by minute 8, patient is cardioverted twice (needs to either increase energy or change pad location on second attempt) AND given two different antiarrhythmics, go to vitals (D). If cardiology is called at this point, nurse lets them know that cardiology is in the catheterization lab and cannot return pages at this time.	B: T 98°F HR 160 BP 130/70 RR 20 O2 sat 98% room air (RA)
8:00	Patient has either been inappropriately defibrillated or given isoproterenol (vitals C). Team has given two different antiarrhythmics and cardioverted twice (vitals D).	Team should recognize ventricular fibrillation and initiate chest compressions. Patient will awaken and have return of spontaneous circulation once defibrillated twice (vitals B). Cardiology is still not available if contacted during ACLS. Team should obtain repeat ECG, note ST-elevation myocardial infarction, then activate the cardiac catheterization lab. Patient will report worsening chest heaviness at this time and will repeat this as needed to obtain post-cardioversion ECG. Cardiology will call back and accept the patient. Case ends.	C: T 98°F HR 180 BP - RR - O2 sat 85% room air (RA) 90% on non-rebreather or 98% on bag-valve mask D: T 98F HR 88 BP 110/60 Resp 20 O2 sat 98%

**Table t11-jetem-8-4-s25:** 

**Critical Actions:** Obtain an early (within first 2 minutes) ECG Request a previous ECG Place defibrillator pads within a minute of reviewing current ECG Perform synchronized cardioversion Administer two different negative inotropes Activate the cardiac catheterization lab	0:00

**Table t12-jetem-8-4-s25:** 

	Milestone	Did not achieve level 1	Level 1	Level 2	Level 3
**1**	**Emergency Stabilization (PC1)**	□ Did not achieve Level 1	□ Recognizes abnormal vital signs	□ Recognizes an unstable patient, requiring intervention Performs primary assessment Discerns data to formulate a diagnostic impression/plan	□ Manages and prioritizes critical actions in a critically ill patient Reassesses after implementing a stabilizing intervention
**2**	**Performance of focused history and physical (PC2)**	□ Did not achieve Level 1	□ Performs a reliable, comprehensive history and physical exam	□ Performs and communicates a focused history and physical exam based on chief complaint and urgent issues	□ Prioritizes essential components of history and physical exam given dynamic circumstances
**3**	**Diagnostic studies (PC3)**	□ Did not achieve Level 1	□ Determines the necessity of diagnostic studies	□ Orders appropriate diagnostic studies. Performs appropriate bedside diagnostic studies/procedures	□ Prioritizes essential testing Interprets results of diagnostic studies Reviews risks, benefits, contraindications, and alternatives to a diagnostic study or procedure
**4**	**Diagnosis (PC4)**	□ Did not achieve Level 1	□ Considers a list of potential diagnoses	□ Considers an appropriate list of potential diagnosis May or may not make correct diagnosis	□ Makes the appropriate diagnosis Considers other potential diagnoses, avoiding premature closure
**5**	**Pharmacotherapy (PC5)**	□ Did not achieve Level 1	□ Asks patient for drug allergies	□ Selects an medication for therapeutic intervention, consider potential adverse effects	□ Selects the most appropriate medication and understands mechanism of action, effect, and potential side effects Considers and recognizes drug-drug interactions
**6**	**Observation and reassessment (PC6)**	□ Did not achieve Level 1	□ Reevaluates patient at least one time during case	□ Reevaluates patient after most therapeutic interventions	□ Consistently evaluates the effectiveness of therapies at appropriate intervals
**7**	**Disposition (PC7)**	□ Did not achieve Level 1	□ Appropriately selects whether to admit or discharge the patient	□ Appropriately selects whether to admit or discharge Involves the expertise of some of the appropriate specialists	□ Educates the patient appropriately about their disposition Assigns patient to an appropriate level of care (ICU/Tele/Floor) Involves expertise of all appropriate specialists
**9**	**General Approach to Procedures (PC9)**	□ Did not achieve Level 1	□ Identifies pertinent anatomy and physiology for a procedure Uses appropriate Universal Precautions	□ Obtains informed consent Knows indications, contraindications, anatomic landmarks, equipment, anesthetic and procedural technique, and potential complications for common ED procedures	□ Determines a back-up strategy if initial attempts are unsuccessful Correctly interprets results of diagnostic procedure
**20**	**Professional Values (PROF1)**	□ Did not achieve Level 1	□ Demonstrates caring, honest behavior	□ Exhibits compassion, respect, sensitivity and responsiveness	□ Develops alternative care plans when patients’ personal beliefs and decisions preclude standard care
**22**	**Patient centered communication (ICS1)**	□ Did not achieve level 1	□ Establishes rapport and demonstrates empathy to patient (and family) Listens effectively	□ Elicits patient’s reason for seeking health care	□ Manages patient expectations in a manner that minimizes potential for stress, conflict, and misunderstanding. Effectively communicates with vulnerable populations, (at risk patients and families)
**23**	**Team management (ICS2)**	□ Did not achieve level 1	□ Recognizes other members of the patient care team during case (nurse, techs)	□ Communicates pertinent information to other healthcare colleagues	□ Communicates a clear, succinct, and appropriate handoff with specialists and other colleagues Communicates effectively with ancillary staff

## INSTRUCTOR MATERIALS

**Case Title:** Ventricular Tachycardia

**Case Description & Diagnosis (short synopsis):** Patient is a 70-year-old male who presents to triage by private vehicle with an hour of weakness and dizziness. Upon further questioning, he reports mild chest heaviness and shortness of breath.

Participants should request an electrocardiogram. Once VT is identified, they should place automated external defibrillator pads and empirically start amiodarone. Savvy providers may note that the VT is polymorphic but not Torsades, indicating possible acute ischemia. The patient is initially hemodynamically stable without hypotension, significant chest pain, signs of decompensated heart failure, or altered mentation. Providers may provide empiric magnesium. A first attempt at synchronized cardioversion will be unsuccessful, and subsequent attempts will only be successful if a second anti-arrhythmic is chosen and cardioversion is troubleshot – whether through changing the location of the pads or increasing the amount of energy delivered. If the patient is inappropriately defibrillated or given isoproterenol, they will develop ventricular fibrillation requiring appropriate advanced cardiovascular life support measures to develop ROSC back to stable VT. Once patient is cardioverted by two different types of anti-arrhythmic and two attempts of synchronized cardioversion, they will convert to sinus. The patient will report worsening chest heaviness and a repeat ECG will demonstrate an inferior STEMI. Cardiology will only return pages once patient is back in sinus rhythm. Providers should describe the patient’s emergency department course and activate the cardiac catheterization lab.

**Equipment or Props Needed:**

- High fidelity simulation mannequin (authors used a SimMan^®^ 3G Manikin)
- Angiocatheters for peripheral intravenous access = 18g, 20g, 22g
- Cardiac monitor
- Pulse oximetry
- IV pole
- Nasal cannula
- Automated external defibrillator with two sets of pads
- Normal saline (1 liter x2)
- Lactated Ringers (1 liter x2)
- Simulated medications with labeling: amiodarone, lidocaine, procainamide, propranolol, metoprolol, isoproterenol, diltiazem, magnesium, aspirin

**Actors needed:**

One actor as the primary nurse. Faculty may call in overhead as the cardiologist (and pharmacist, if desired).

**Stimulus Inventory:** [Table t2-jetem-8-4-s25]

**Background and brief information:** Patient is a 70-year-old male who presents to your ED via triage with dizziness and generalized weakness for the past hour.

**Initial presentation:** 70-year-old male lying supine on the cot, conversant

- Past medical history: type 2 diabetes mellitus, hypertension, hyperlipidemia
- Past surgical history: none
- Medications: metformin, lisinopril, simvastatin
- Allergies: none
- Social hx: smokes cigarettes. Denies alcohol or illicit drug use
- Family history: both parents had coronary artery disease and high blood pressure
- Vital signs:
  ○ HR – 160 beats per minute
  ○ Resp rate – 20 respirations per minute
  ○ Temp – 98° F
  ○ BP – 130/70 mmHg
  ○ Pulse ox – 98% on room air
- Weight: 80 kilograms (kg)

Assessment: Lying supine, conversational

**How the scene unfolds:** Patient is a 70-year-old male who presents with steadily progressing ischemic chest discomfort and a wide complex tachydysrhythmia secondary to an underlying inferior STEMI, which is hidden unless the patient is successfully converted to a sinus rhythm.

Patient’s vitals are initially stable. Participants should request an ECG and correctly interpret it as VT. At that point, a previous ECG should be requested, the patient should be put on a cardiac monitor with defibrillator pads placed, and amiodarone bolus administered. Patient may be treated empirically with intravenous magnesium. Participants will discuss synchronized cardioversion as well as administration of other agents such as lidocaine. If electrical storm is identified, participants can also administer beta blockers, propofol, or shock again with an increase in delivered voltage or change in pad placement. Participants have eight minutes to identify the dysrhythmia, administer two negative inotropic medications, and administer two synchronized cardioversions. If participants are unable to do this or defibrillate, the rhythm degenerates into ventricular fibrillation. After one round of ACLS including shocks, the patient will convert and an inferior STEMI can be seen on the post-conversion ECG. If participants call cardiology too early before the medications are given, shocks are delivered, or during a ventricular fibrillation arrest, cardiology will be busy. The encounter ends after providers activate the catheterization lab and can speak with a cardiologist. The patient should ultimately be dispositioned to the medical intensive care unit or coronary care unit.

**Critical actions:**

1. Obtain an early (within first 2 minutes) ECG
2. Request a previous ECG
3. Place defibrillator pads within a minute of reviewing current ECG
4. Perform synchronized cardioversion
5. Administer two different negative inotropes
6. Activate the cardiac catheterization lab

**Case Title:** Ventricular Tachycardia

**Chief Complaint:** Weakness and dizziness[Table t3-jetem-8-4-s25]

**General Appearance:** Lying supine in the cot, conversational.

**Primary Survey:**

- **Airway:** patent
- **Breathing:** clear to auscultation bilaterally
- **Circulation:** heart regular rhythm, tachycardic rate. 2+ distal symmetric pulses

**History:**

- **History of present illness:** Patient reports an hour of weakness and dizziness which has been constant. He has mild chest discomfort and shortness of breath with nausea.
  If asked:
  ○ He was doing yardwork an hour ago when symptoms began
  ○ Symptoms are worsened with exertion and improved with rest
  ○ The dizziness is presyncope without vertiginous component. There has not been any syncope
  ○ He has never had a stress test, heart catheterization, or echocardiogram
  ○ No family history of dysrhythmias, syncope, or sudden cardiac death
- **Past medical history:** type 2 diabetes mellitus, hypertension, hyperlipidemia
- **Past surgical history:** none
- **Medications:** metformin, lisinopril, simvastatin
- **Allergies:** none
- **Social history:** smokes cigarettes. Denies alcohol or illicit drug use
- **Family history:** both parents had coronary artery disease and high blood pressure

**Vital Signs:**

- HR – 160 beats per minute
- Resp rate – 20 respirations per minute
- Temp – 98° Fahrenheit
- BP – 130/70
- Pulse ox – 98% on room air
- Weight: 80 kg

**Assessment:** Lying supine on the cot, conversational

**Secondary Survey/Physical Examination:**

- **General Appearance**: lying supine on cot, appears stated age, no acute cardiopulmonary distress but reports mild chest discomfort, dyspnea, nausea when asked
- **HEENT**
  ○ Head: within normal limits
  ○ Eyes: within normal limits
  ○ Ears: within normal limits
  ○ Nose: within normal limits
  ○ Throat/oropharynx: within normal limits
- **Neck**: No jugular venous distention
- **Heart**: regular rhythm, tachycardic rate. No rubs, murmurs or gallops. S1, S2+ symmetric distal pulses
- **Lungs**: clear to auscultation bilaterally, no rhonchi, wheezes, or rales. Able to speak in complete sentences without accessory muscle use
- **Abdominal/GI**: within normal limits
- **Genitourinary**: deferred/within normal limits
- **Rectal**: deferred/within normal limits
- **Extremities**: No lower extremity edema
- **Neuro**: within normal limits
- **Skin**: within normal limits. No diaphoresis.
- **Lymph**: within normal limits
- **Psych**: within normal limits

*Electrocardiogram, VT*

Author’s own image[Fig f1-jetem-8-4-s25]

*Electrocardiogram, previous, sinus rhythm*

Glen Larson. 12 lead generated sinus rhythm. In: Wikimedia Commons.

https://commons.wikimedia.org/wiki/File:12_lead_generated_sinus_rhythm.JPG. Public domain. Published 25 December 2006.[Fig f2-jetem-8-4-s25]

*Electrocardiogram, post conversion, ST-elevation myocardial infarction*

Andrew Meyerson. ST Segment Elevation Myocardial Infarction Unlabeled. In: Wikimedia Commons.

https://commons.wikimedia.org/wiki/File:ST_Segment_Elevation_Myocardial_Infarction_Unlabeled.jpg. Public domain. Published 19 May 2014.[Fig f3-jetem-8-4-s25]

*Chest X-ray*

Stillwaterising. Chest Xray PA 3-8-2010. In: Wikimedia Commons. File:Chest Xray PA 3-8-2010.png - Wikimedia Commons. Public domain. Published 8 March 2010.[Fig f4-jetem-8-4-s25][Table t4-jetem-8-4-s25][Table t5-jetem-8-4-s25][Table t6-jetem-8-4-s25][Table t7-jetem-8-4-s25][Table t8-jetem-8-4-s25][Table t9-jetem-8-4-s25]

## OPERATOR MATERIALS

[Table t10-jetem-8-4-s25]

**Diagnosis:**

VT secondary to acute ischemia

**Disposition:**

Cardiac catheterization lab

## DEBRIEFING AND EVALUATION PEARLS

### Ventricular Tachycardia

**A formal debrief was conducted with the following objectives:**

- Closed loop communication and team interaction
  ○ Pointed roles during a critical event are extremely important in providing maximal care
  ○ Address team members by their roles or names when asking for an action and request a verbal confirmation that you were heard and understood
- Definition of VT and electrical storm
- Types of VT and underlying causes
- General management of VT
- How the QT and QTc Interval are calculated
- Specific management of:
  ○ Monomorphic VT
  ○ Polymorphic VT without prolonged QTc
  ○ Torsades de Pointes
- Medication choices for refractory VT
- Indications to activate catheterization lab

The debrief included a general overview of the scenario. The debrief focused not on mistakes or outcomes of the case, but rather the general perspective of the case and what led them to make the decisions that they did during the scenario.

We started the main discussion by asking what participants thought the primary diagnosis was and walking them through a diagnosis of electrical storm. Electrical storm may be defined as three or more episodes of sustained VT, sustained ventricular fibrillation, or appropriate implantable cardioverter-defibrillator shocks within a 24-hour period requiring termination by an intervention such as cardioversion, anti-tachycardia pacing or defibrillation.^1^,^3^ We explain that the secondary goal of the case was pharmacologic management, which is why the simulation patient did not convert with just one dose of an antiarrhythmic or negative inotropic medication. Common causes of storm were discussed including: nonischemic cardiomyopathy, arrhythmogenic right ventricular cardiomyopathy, sarcoidosis, amyloidosis, Chagas disease, and Brugada syndrome.^2^

We organized VT into three categories - monomorphic VT, generic polymorphic VT (polymorphic VT with a regular QTc) and Torsades de Pointes.^5^ Management of each of these was discussed; however, generic polymorphic VT was the case that was emphasized the most since this was the diagnosis of the patient.

The primary learning point was that polymorphic VT without a prolonged QTc is most commonly due to acute ischemia.^1^

We discussed that monomorphic VT is usually due to structural heart disease from prior MI.^1^ Both monomorphic and polymorphic VT without a prolonged QTc are medically managed similarly with trials of amiodarone, procainamide, lidocaine, as well as identifying and addressing underlying causes.

Torsades is polymorphic VT with a prolonged QTc and needs to be managed with electricity, magnesium, isoproterenol, or overdrive pacing.^6^

During the discussion of Torsades, we discussed the QT/QTc and the QT Interval nomogram.^7^ We emphasized how to calculate the QTc or take note if a transition can be identified on ECG or telemetry.

Emphasis was made that if the incorrect medications were administered, such as rate controlling a Torsades patient or increasing the rate of a patient with ischemia, deleterious results may occur.

The next major points of discussion covered general management of all types of VT. In general, it would not be; incorrect to order magnesium for patients in VT, especially in Torsades or those with a prolonged QTc. It is important to note that serum magnesium often does not reflect the true intracellular concentrations.^8^ Correcting electrolyte abnormalities was discussed. As for medications, we discussed amiodarone, lidocaine, procainamide, isoproterenol as well as beta-blockers including propranolol, esmolol and metoprolol. We focused our attention on amiodarone, lidocaine and esmolol because at our institution, these medications can be readily obtained in our medication dispensing cabinet. Compared to lidocaine, amiodarone has shown to have improved survival to hospital admission for shock-resistant VT or ventricular fibrillation.^9^ However, there is no further robust evidence to definitively support one anti-arrhythmic over another at this time.

Special mention was made for propofol with subsequent intubation, stellate ganglion nerve blocks since the left stellate ganglia provides most of the myocardium’s sympathetic innervation,^10^ and double sequential external defibrillation to increase delivered energy and/or depolarize the myocardium along a different vector,^10^,^11^ but these treatments were not discussed in detail.

We concluded the debrief session discussing indications for catheterization lab including occlusive myocardial infarction, NSTEMI/unstable angina refractory to medical therapy, cardiogenic shock, electrical storm and unstable bradydysrhythmias.

## SIMULATION ASSESSMENT

*Ventricular Tachycardia*

Learner: _________________________________________

***Assessment Timeline***

This timeline is to help observers assess their learners. It allows observer to make notes on when learners performed various tasks, which can help guide debriefing discussion.[Table t11-jetem-8-4-s25]

**Critical Actions:**

□ Obtain an early (within first 2 minutes) ECG
□ Request a previous ECG
□ Place defibrillator pads within a minute of reviewing current ECG
□ Perform synchronized cardioversion
□ Administer two different negative inotropes
□ Activate the cardiac catheterization lab

**Summative and formative comments:**

**Milestones assessment:** [Table t12-jetem-8-4-s25]
