# Supplementary material for: Ventricular Tachycardia
Source: J Educ Teach Emerg Med. 2023 Oct 31;8(4):S25–48. doi: 10.21980/J8KD2R (PMC10631810; doi:10.21980/J8KD2R)
Supplement: Supplementary file 1 [file jetem-8-4-s25-supp1.pptx]

## Slide 1
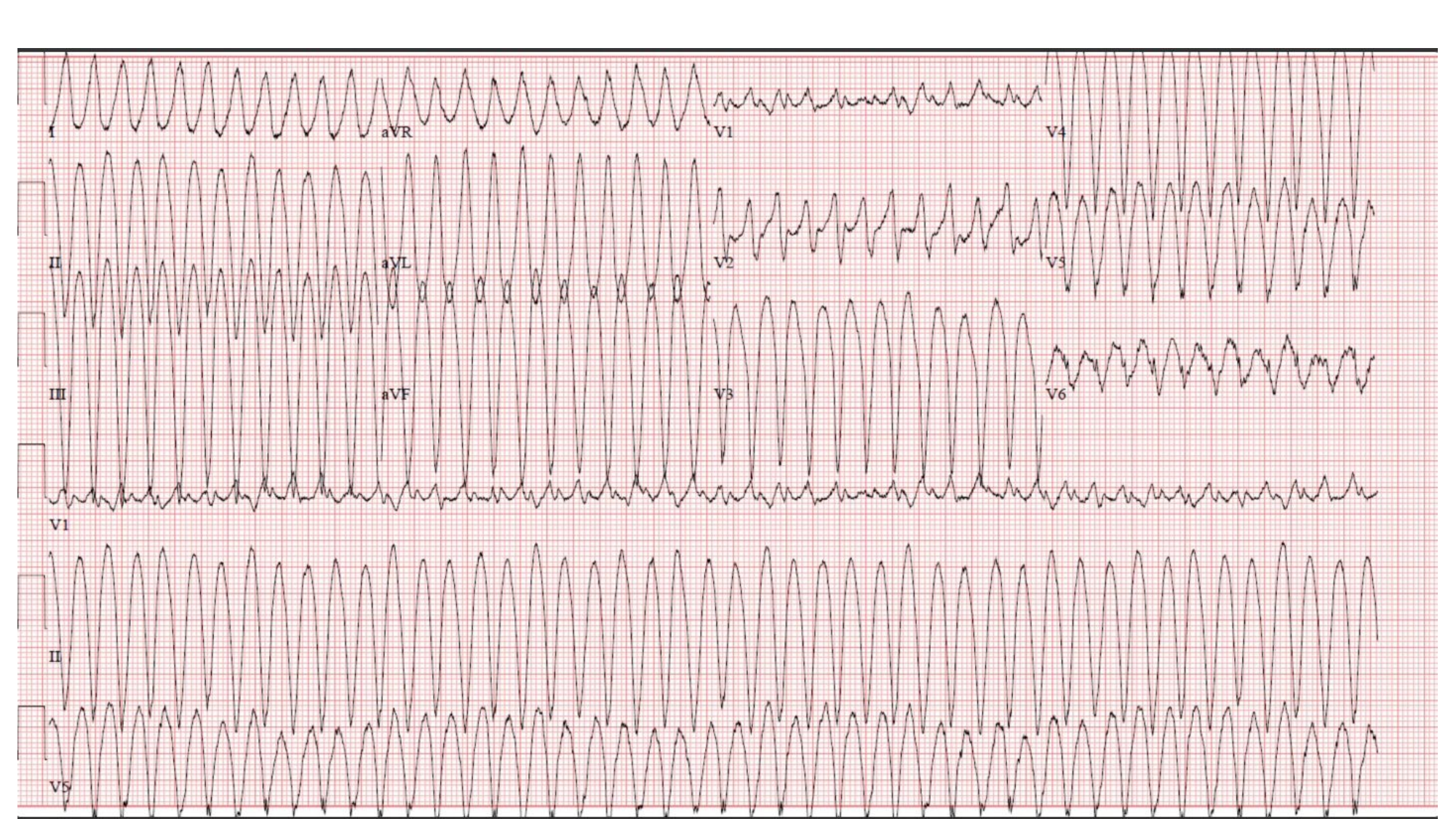

## Slide 2
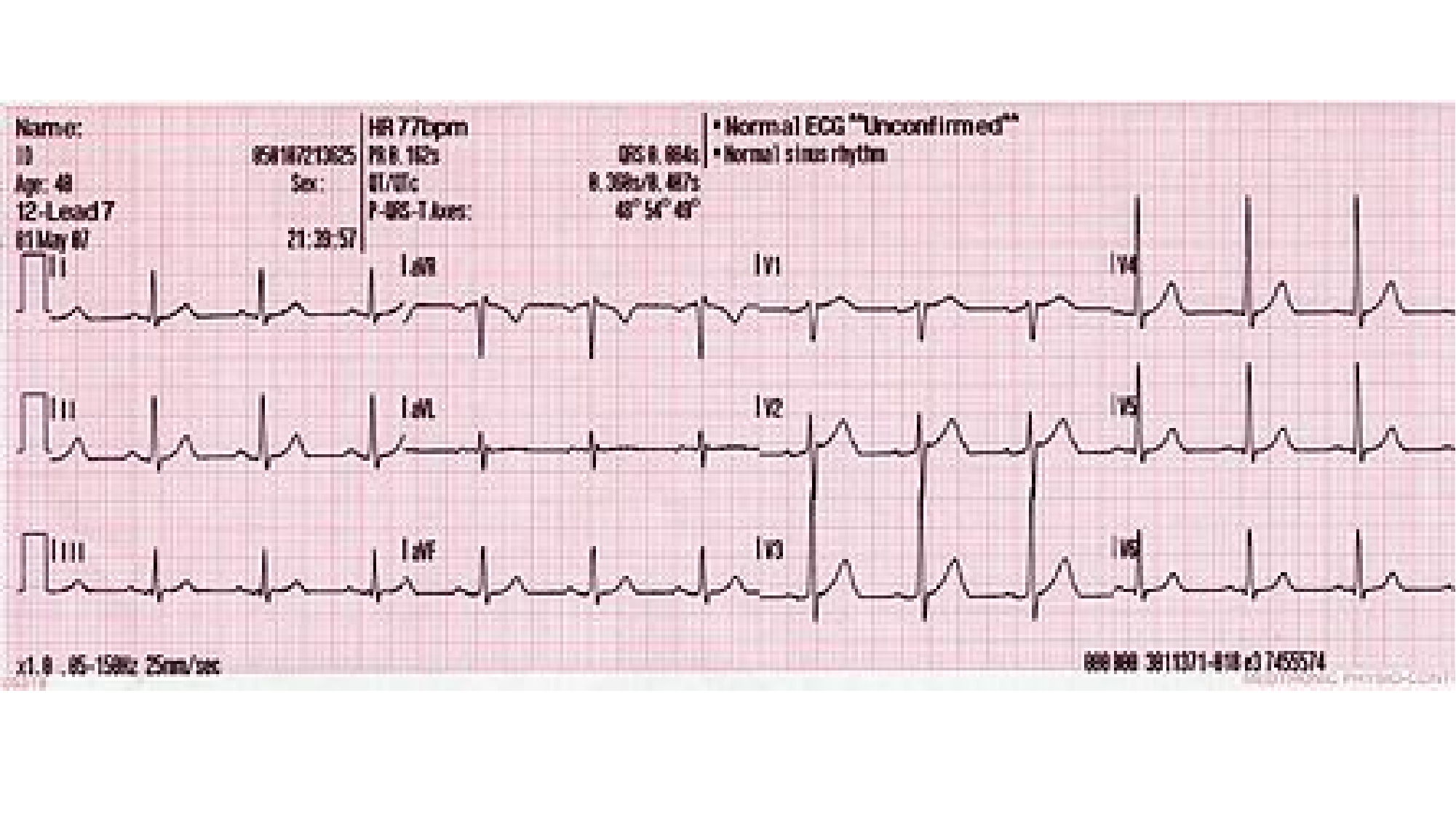

## Slide 3
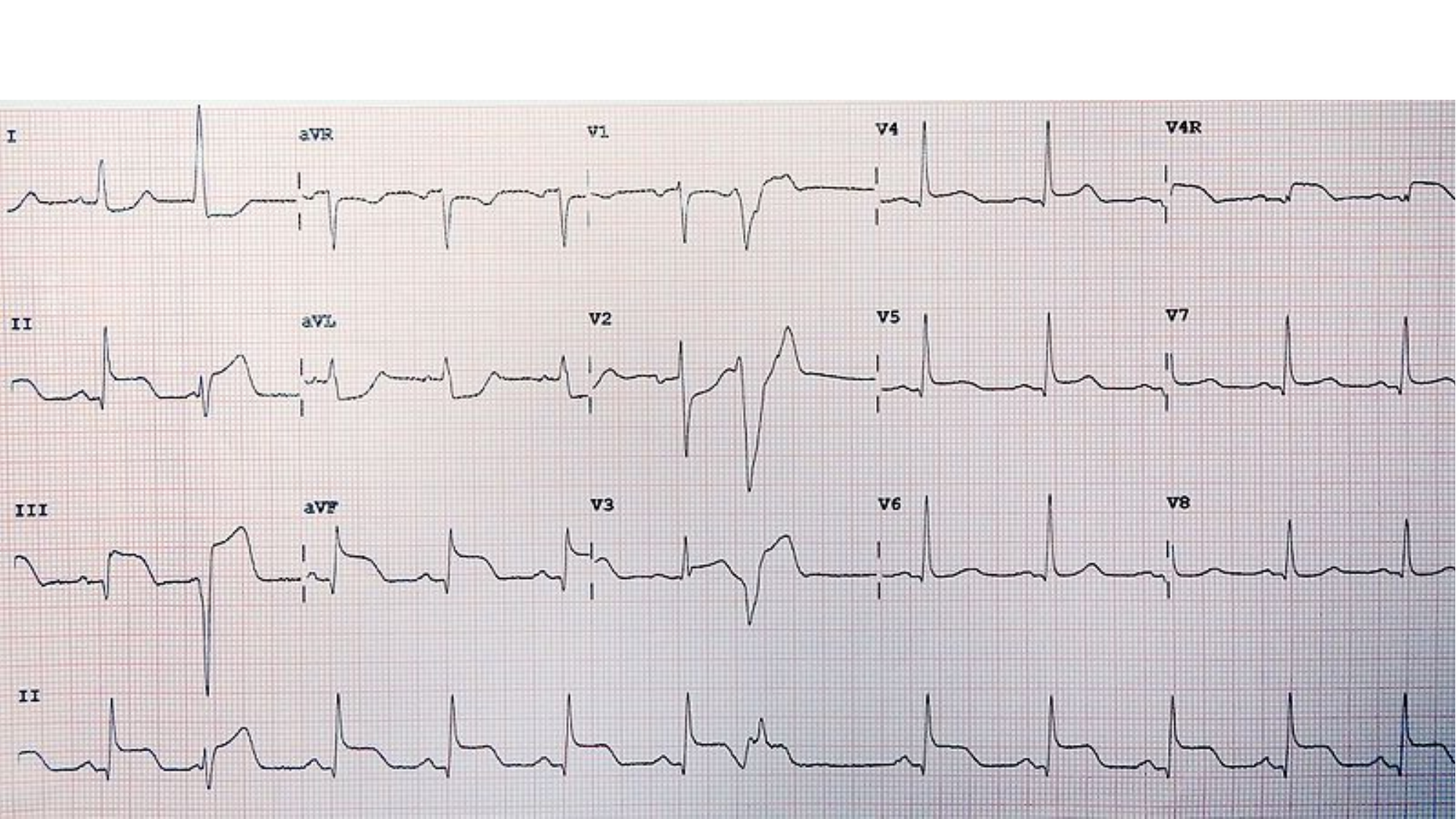

## Slide 4
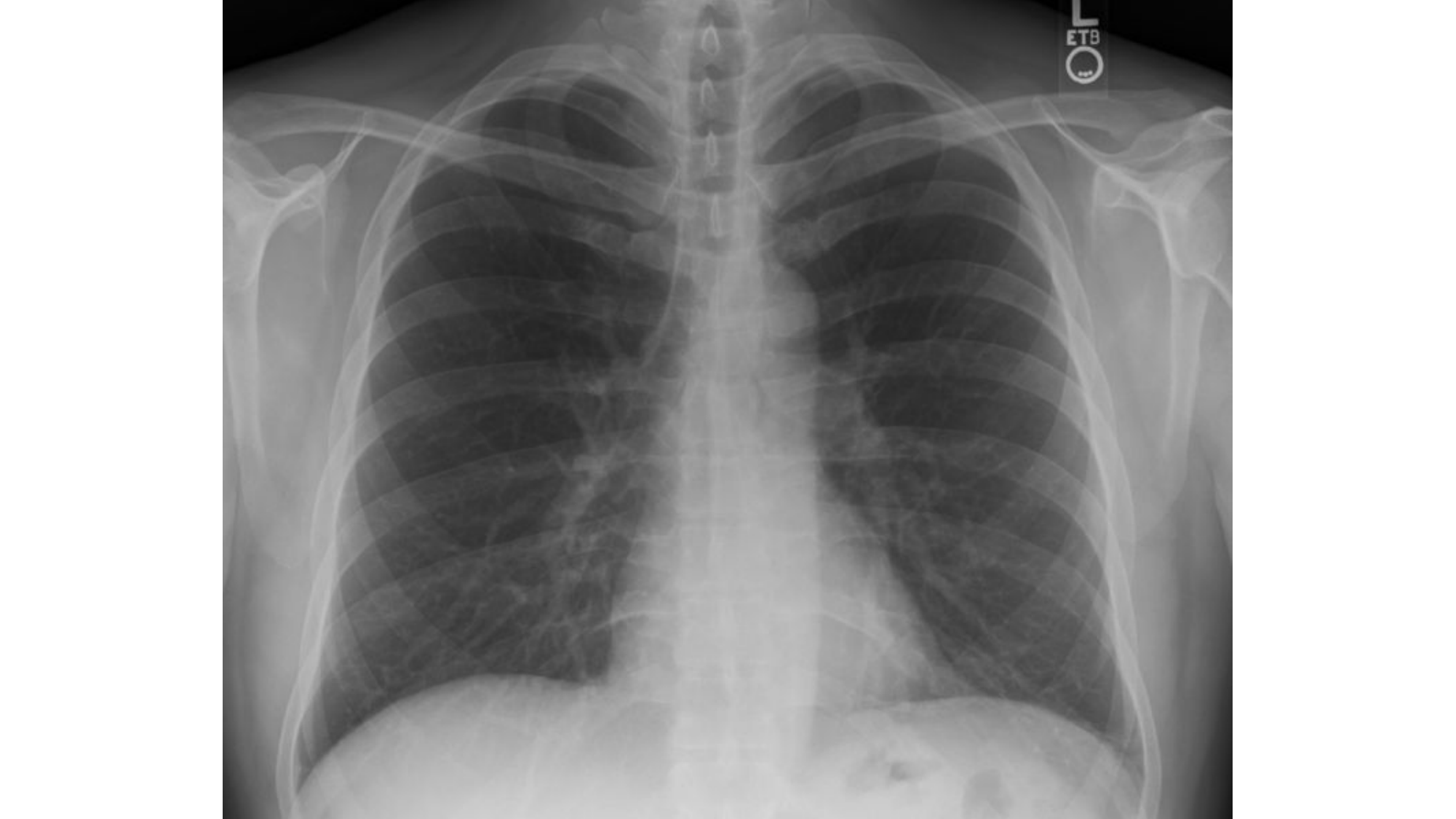

## Slide 5
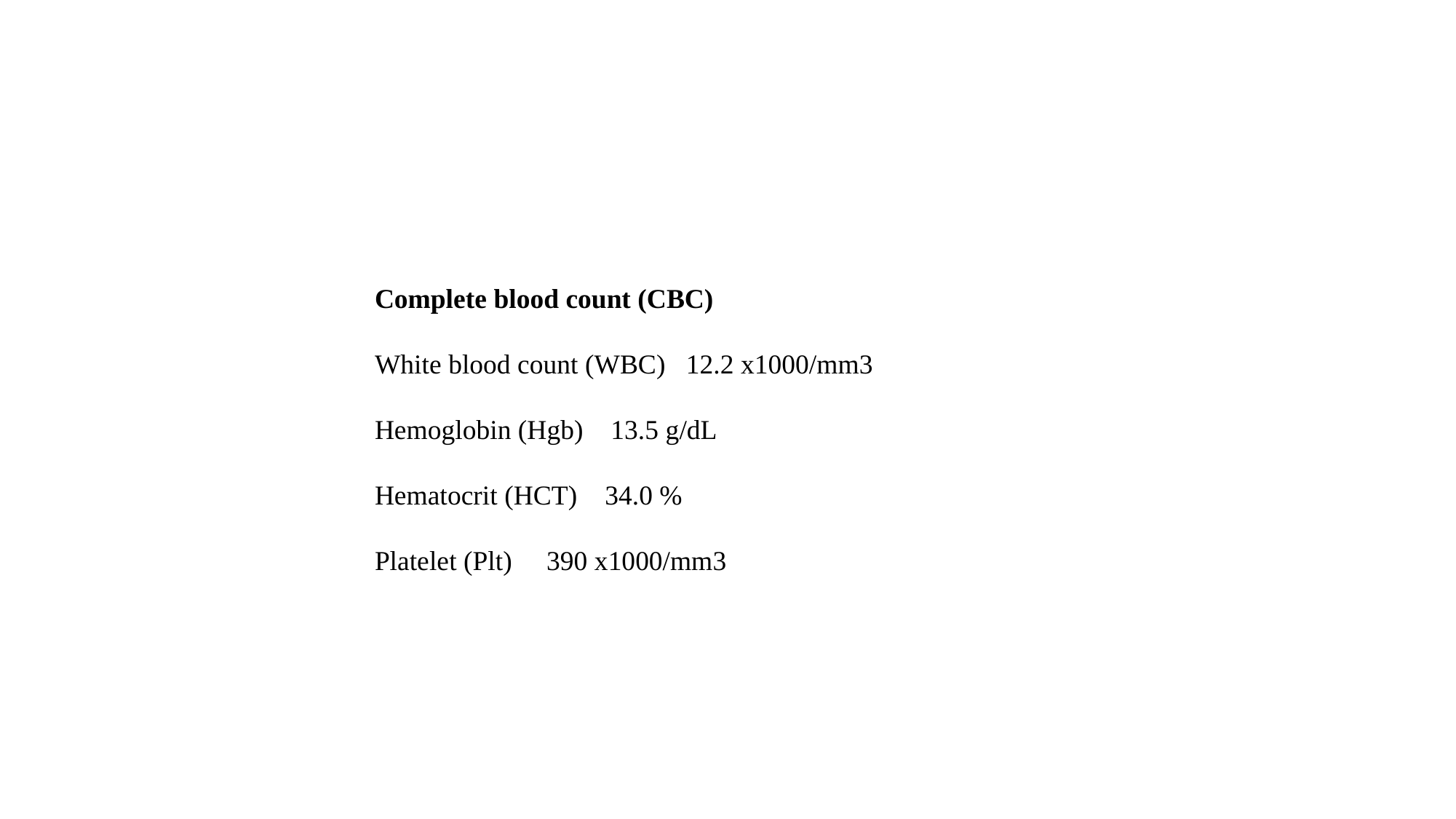

Complete blood count (CBC)
White blood count (WBC) 12.2 x1000/mm3
Hemoglobin (Hgb) 13.5 g/dL
Hematocrit (HCT) 34.0 %
Platelet (Plt) 390 x1000/mm3

## Slide 6
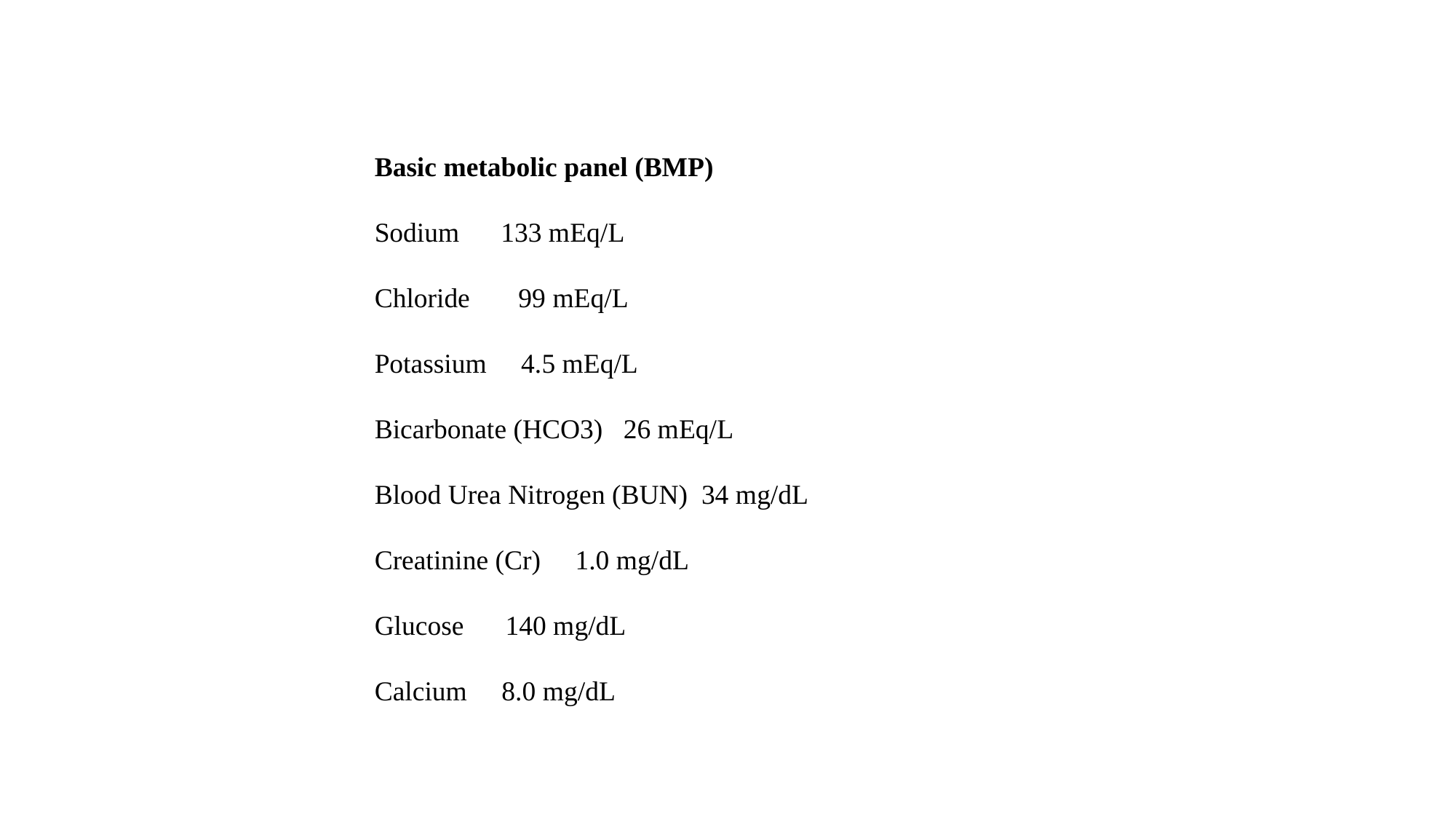

Basic metabolic panel (BMP)
Sodium 133 mEq/L
Chloride 99 mEq/L
Potassium 4.5 mEq/L
Bicarbonate (HCO3) 26 mEq/L
Blood Urea Nitrogen (BUN) 34 mg/dL
Creatinine (Cr) 1.0 mg/dL
Glucose 140 mg/dL
Calcium 8.0 mg/dL

## Slide 7
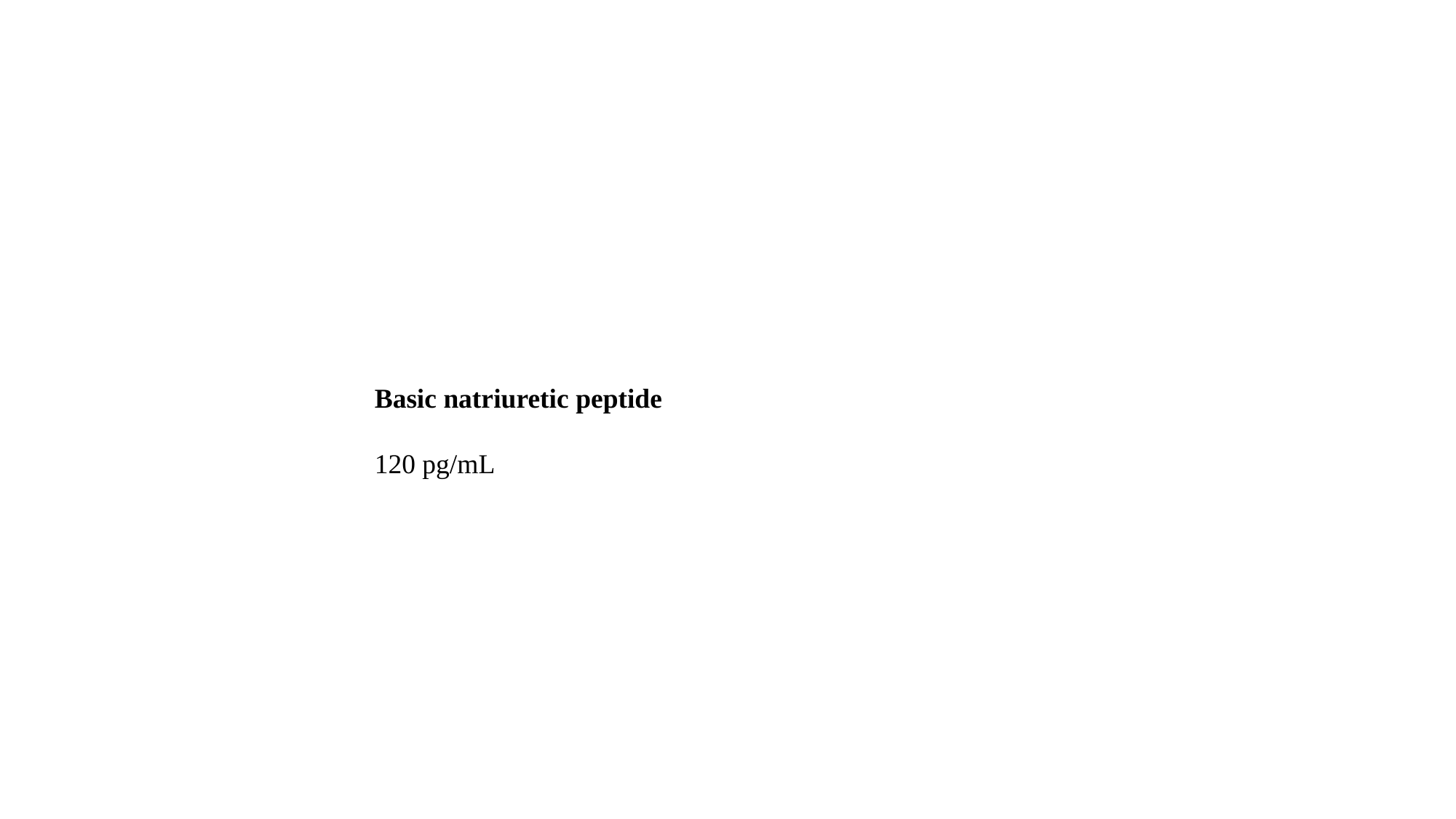

Basic natriuretic peptide
120 pg/mL

## Slide 8
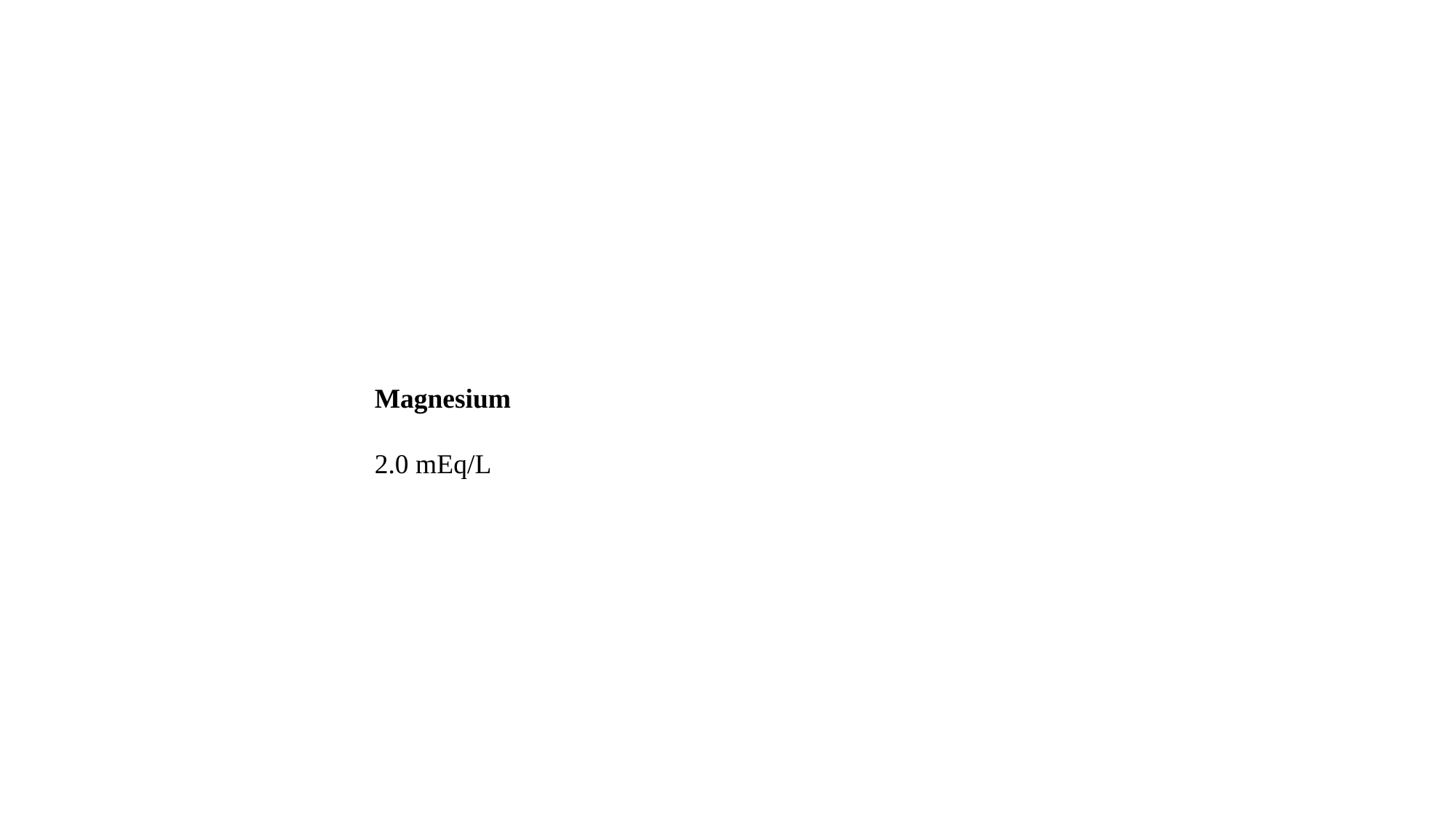

Magnesium
2.0 mEq/L

## Slide 9
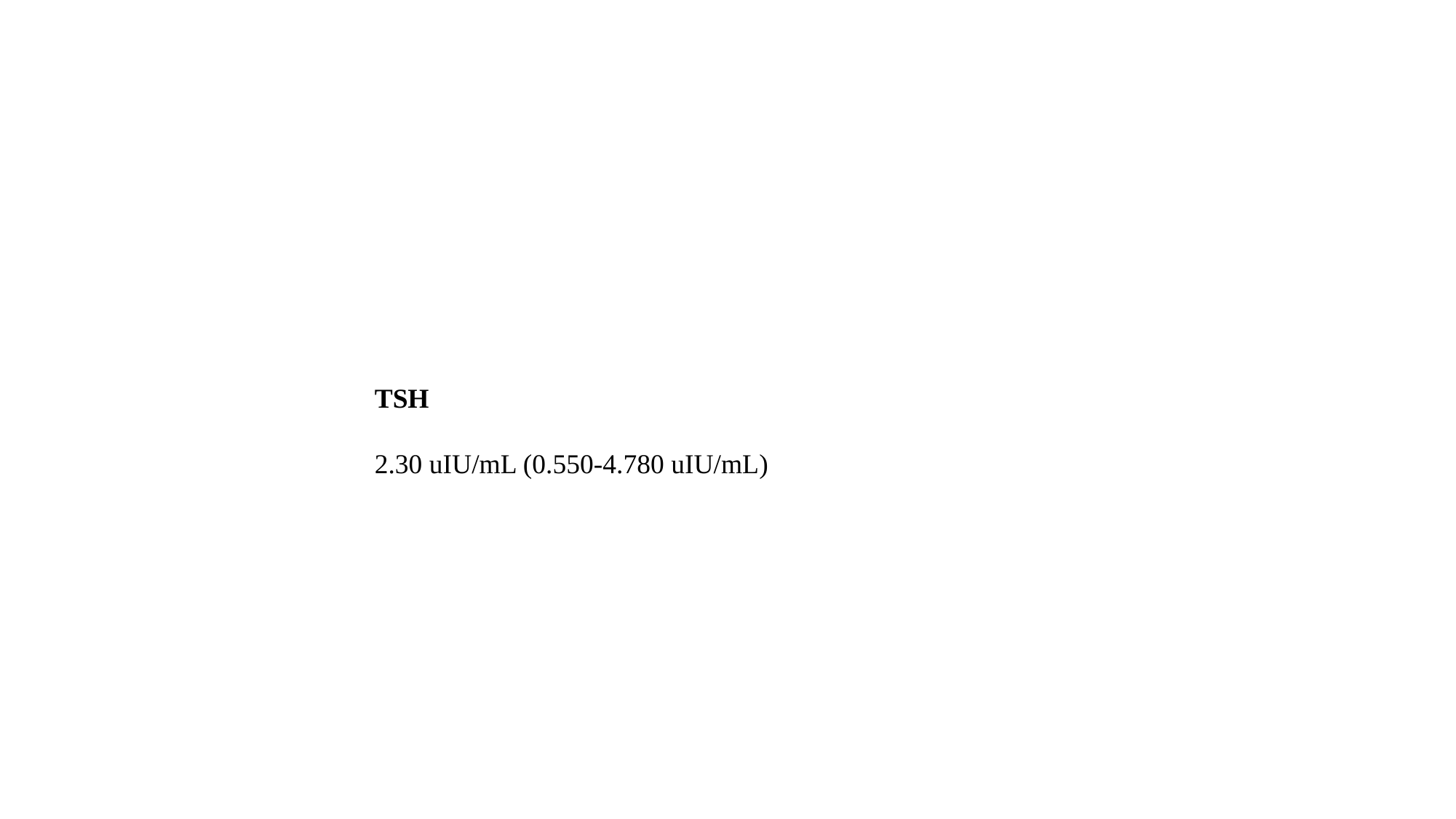

TSH
2.30 uIU/mL (0.550-4.780 uIU/mL)

## Slide 10
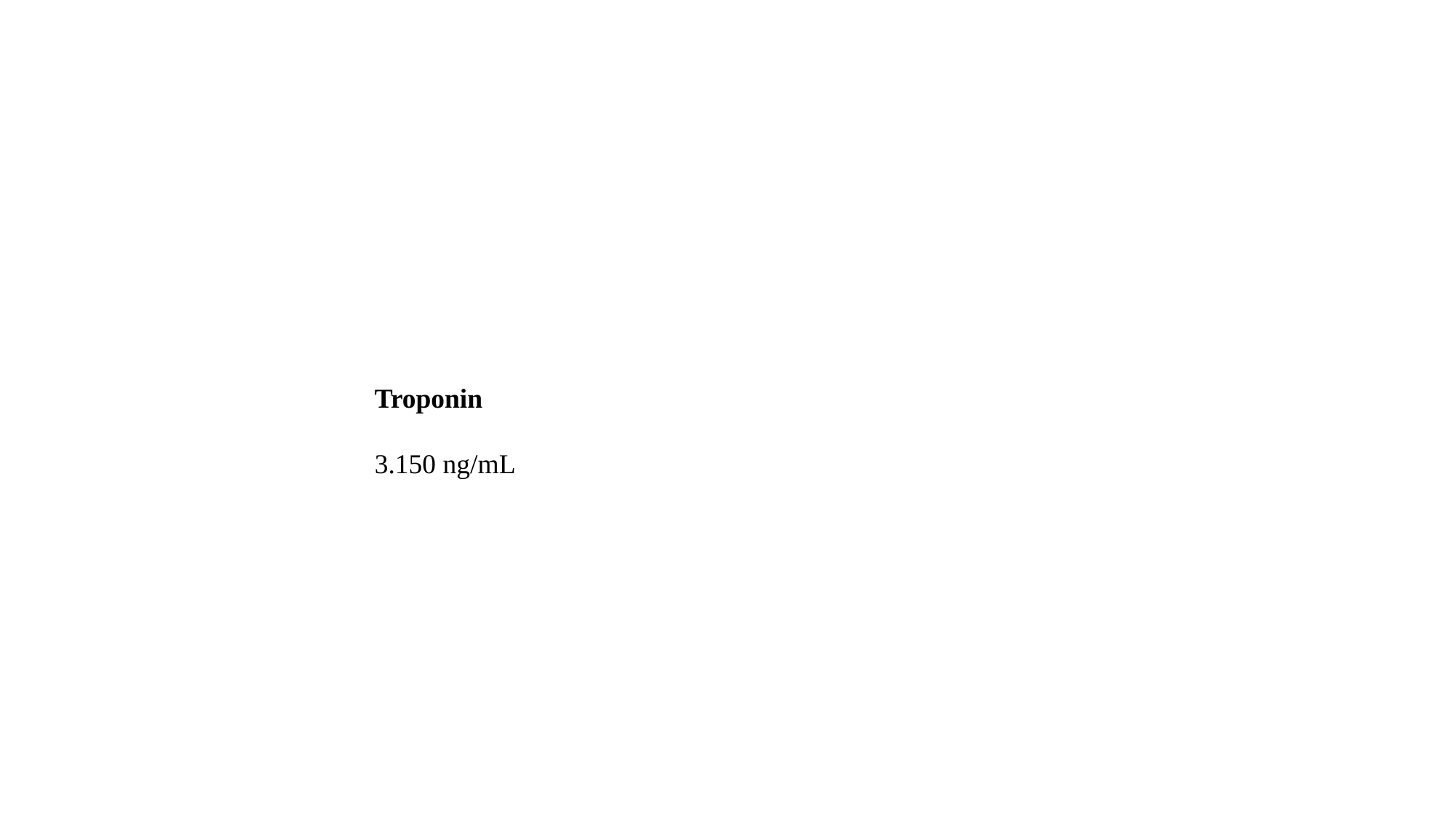

Troponin
3.150 ng/mL
